# Cholangioscopy-directed basket extraction of bile duct stones in a pregnant patient

**DOI:** 10.1055/a-2107-2735

**Published:** 2023-07-13

**Authors:** Jordan Burlen, Anna Cecilia Amaral, Waleed K. Hussain, Samuel Han

**Affiliations:** Division of Gastroenterology, Hepatology, and Nutrition, The Ohio State University Wexner Medical Center, Columbus, OH 43210

Gallstone-related disease and complications are common in pregnancy. Complications of gallstone disease are associated with increased mortality for both the patient and the fetus 1 . Endoscopic retrograde cholangiopancreatography (ERCP) offers a safe and effective treatment for biliary obstruction during pregnancy 2 . Though radiation exposure of ERCP is low, the long-term fetal effects of ERCP-related radiation exposure are unknown 3 . Given the advances in technology and devices for cholangioscopy, we describe an endoscopic technique for cholangioscopy-guided removal of bile duct stones in pregnant patients without the use of any fluoroscopy.

An 18-year-old woman (gravida 1, parity 0) at 30 weeks’ gestation presented with acute gallstone pancreatitis. Magnetic resonance cholangiopancreatography (MRCP) demonstrated cholelithiasis and numerous bile duct stones including an impacted stone at the ampulla ( Fig. 1 ). As the patient was adamant about avoiding any radiation exposure, the use of cholangioscopy without fluoroscopy was offered. Upon duodenal intubation, a bulging papilla with an impacted stone was seen ( Fig. 2 ). A precut sphincterotomy was performed using a free-hand technique, leading to immediate release of the stone ( Fig. 3 ) and facile wire-guided biliary cannulation. Bile aspiration using the sphincterotome confirmed biliary access and a sphincterotomy extension was performed to facilitate cholangioscopy and stone removal. Cholangioscopy (SpyScope DS II, Boston Scientific, Marlborough, Massachusetts, USA) revealed numerous stones within the bile duct ( Video 1 ). A cholangioscopy-directed retrieval basket (SpyBasket, Boston Scientific) was inserted through the cholangioscope with release of the basket upstream of the stone. The basket was then withdrawn with gradual closure allowing for capture of the individual stones ( Fig. 4 ) 4 . The cholangioscope was then withdrawn from the bile duct and each stone released into the duodenum. In total, we retrieved 22 stones ( Fig. 5 ) and confirmed stone clearance with cholangioscopy into the intrahepatic bile ducts. The patient recovered uneventfully and had a healthy delivery 2 months later.

**Fig. 1:**
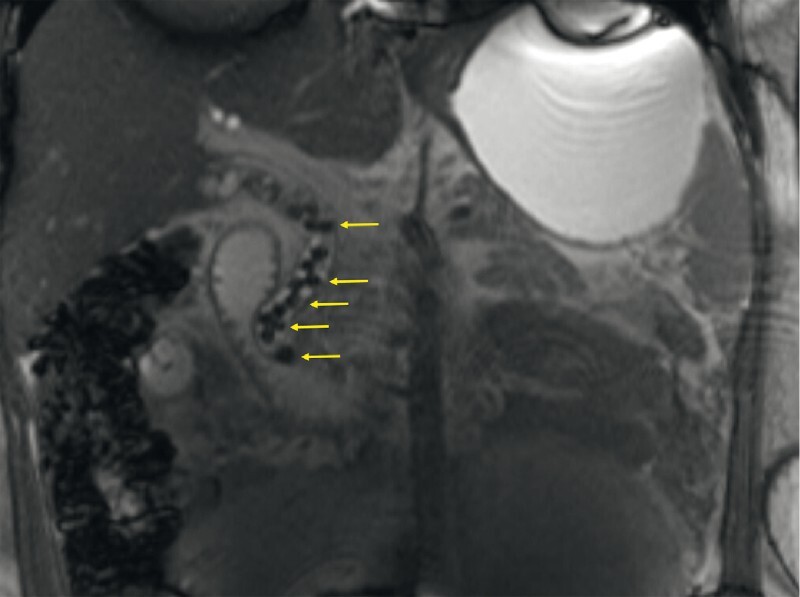
Demonstration of numerous bile duct stones (arrows) on magnetic resonance cholangiopancreatography.

**Fig. 2:**
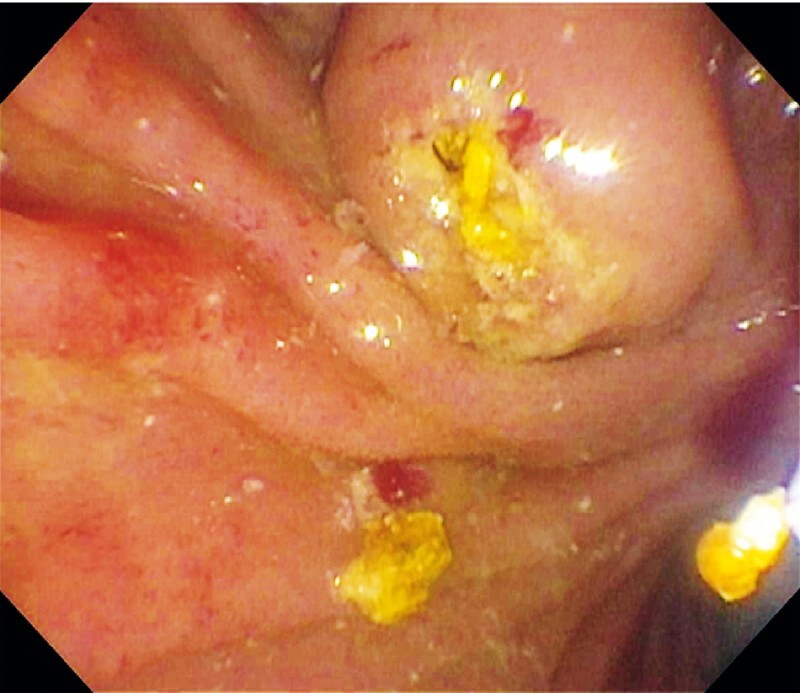
Needle knife precut sphincterotomy to free an impacted stone at the ampulla.

**Fig. 3:**
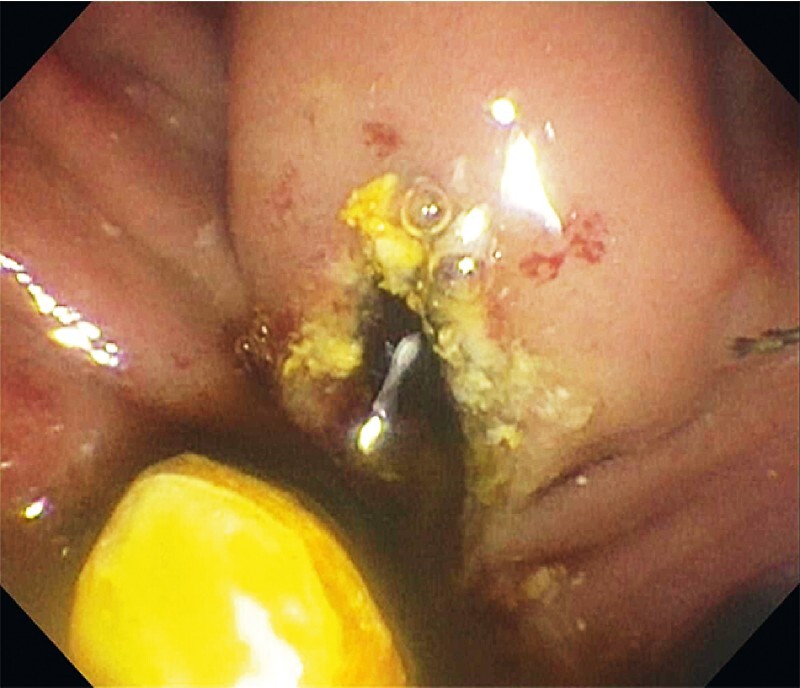
Bile duct stone dislodged from ampulla after precut sphincterotomy.

**Video 1** Cholangioscopy-guided basket retrieval of bile duct stones in a pregnant patient.


**Fig. 4:**
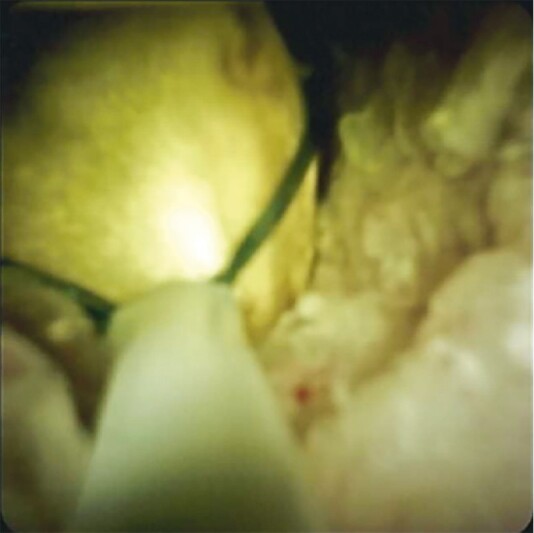
Capture of bile duct stone with a cholangioscopy-directed retrieval basket.

**Fig. 5:**
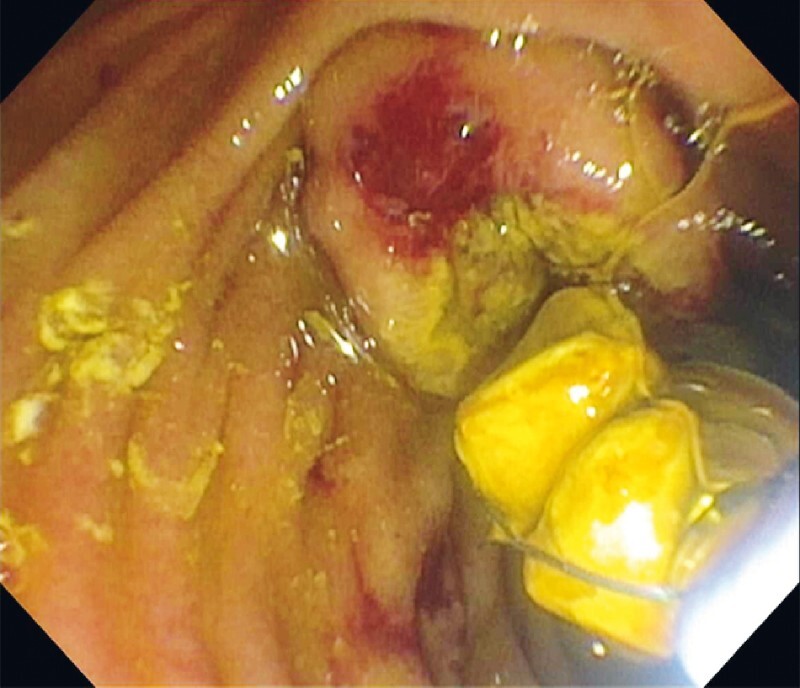
Removal of two bile duct stones with the cholangioscopy-directed retrieval basket.

Endoscopy_UCTN_Code_TTT_1AR_2AH

## Notes

Endoscopy E-Videos

https://eref.thieme.de/e-videos
*E-Videos* is an open access online section of the journal *Endoscopy* , reporting on interesting cases and new techniques in gastroenterological endoscopy. All papers include a high-quality video and are published with a Creative Commons CC-BY license. Endoscopy E-Videos qualify for HINARI discounts and waivers and eligibility is automatically checked during the submission process. We grant 100% waivers to articles whose corresponding authors are based in Group A countries and 50% waivers to those who are based in Group B countries as classified by Research4Life (see: https://www.research4life.org/access/eligibility/ ). This section has its own submission website at https://mc.manuscriptcentral.com/e-videos

## References

[1] Hess E Thumbadoo R P Thorne E Gallstones in pregnancy Br J Hosp Med (Lond) 2021 82 1 8 10.12968/hmed.2020.0330 33646023

[2] Shergill A K Ben-Menachem T Chandrasekhara V Guidelines for endoscopy in pregnant and lactating women Gastrointest Endosc 2012 76 18 24 10.1016/j.gie.2012.02.029 22579258

[3] Sethi S Thosani N Banerjee S Radiation-free ERCP in pregnancy: A “sound” approach to leaving no stone unturned Dig Dis Sci 2015 60 2604 2607 10.1007/s10620-014-3502-y 25577267

[4] Han S Shah R J Cholangioscopy-guided basket retrieval of impacted stones VideoGIE 2020 5 387 388 10.1016/j.vgie.2020.04.009 32954095 PMC7482096

